# Ubiquitination in the regulation of inflammatory cell death and cancer

**DOI:** 10.1038/s41418-020-00708-5

**Published:** 2021-01-11

**Authors:** Peter E. Cockram, Matthias Kist, Sumit Prakash, Si-Han Chen, Ingrid E. Wertz, Domagoj Vucic

**Affiliations:** 1 Departments of Discovery Oncology, Genentech, 1 DNA Way, South San Francisco, CA 94080 USA; 2 Departments of Discovery Chemistry, Genentech, 1 DNA Way, South San Francisco, CA 94080 USA; 3 Departments of Early Discovery Biochemistry, Genentech, 1 DNA Way, South San Francisco, CA 94080 USA

**Keywords:** Ubiquitins, Tumour-suppressor proteins, Inflammasome

## Abstract

The ubiquitin system is complex, multifaceted, and is crucial for the modulation of a vast number of cellular processes. Ubiquitination is tightly regulated at different levels by a range of enzymes including E1s, E2s, and E3s, and an array of DUBs. The UPS directs protein degradation through the proteasome, and regulates a wide array of cellular processes including transcription and epigenetic factors as well as key oncoproteins. Ubiquitination is key to the dynamic regulation of programmed cell death. Notably, the TNF signaling pathway is controlled by competing ubiquitin conjugation and deubiquitination, which governs both proteasomal degradation and signaling complex formation. In the inflammatory response, ubiquitination is capable of both activating and dampening inflammasome activation through the control of either protein stability, complex formation, or, in some cases, directly affecting receptor activity. In this review, we discuss the enzymes and targets in the ubiquitin system that regulate fundamental cellular processes regulating cell death, and inflammation, as well as disease consequences resulting from their dysregulation. Finally, we highlight several pre-clinical and clinical compounds that regulate ubiquitin system enzymes, with the aim of restoring homeostasis and ameliorating diseases.

## Graphical abstract

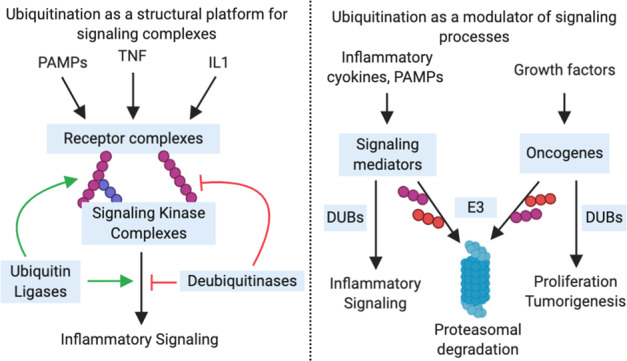

## Facts

- Signaling pathways activated by TNF are intricately controlled by opposing ubiquitination and deubiquitination thus allowing precise spacial and temporal activation of signaling complexes.
- Inflammatory responses utilize ubiquitination to carefully regulate protein activity and stability for the optimal inflammasome activation.
- Ubiquitin signaling can be genetically dysregulated in human disease and during infection by pathogens. Targeting such dysregulation is of therapeutic value to combat these diseases.
- An increasing number of targeted therapeutics are being developed that are enabling safer and more efficacious treatment regimens, particularly when dosed in combination.

## Open questions

- Why are many signaling proteins ubiquitinated without a clear functional relevance for the signaling outcome(s)?
- How do immunoregulatory drugs elicit their anti-inflammatory effect through CRBN-dependent and -independent mechanisms?
- An efficacious frontline therapeutic regimen for patients with multiple myeloma includes treatment with IMiDs, which achieve therapeutic benefit by promoting the proteasomal degradation of critical cellular targets, combined with proteasome inhibitors. Mechanistically, how do these seemingly opposing treatment strategies achieve therapeutic benefit?

## Introduction—the ubiquitin system

**Fig. 1:**
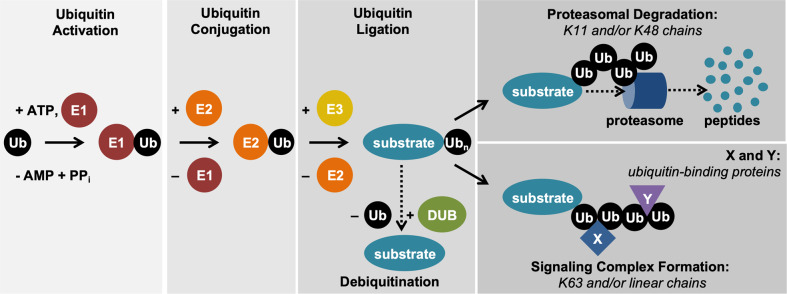
Ubiquitin proteasome system. Ubiquitination is a multistep process that involves ubiquitin activation by E1 enzymes, ubiquitin conjugation to E2 enzymes, and ubiquitin ligation to the substrate protein via E3 enzymes. Ubiquitination can result in proteasomal degradation of the substrate or in recruitment of the substrate to multiprotein complexes, depending on the topology of the polyubiquitin chain linkages. X and Y indicate ubiquitin chain-binding proteins.

Ubiquitination is an essential posttranslational modification that covalently links the 76-amino acid ubiquitin protein to a target protein. The reaction is a multistep process mediated by three classes of enzymes: ubiquitin activating enzymes (E1), ubiquitin conjugating enzymes (E2), and ubiquitin ligases (E3) [1] (Fig. 1). E1s activate and transfer ubiquitin to E2s, while E3s recruit the substrate proteins for the transfer of ubiquitin moieties. E1 and E2 families are relatively small with 2 and 42 members, respectively, whereas several hundred ubiquitin ligases have been identified [2, 3]. Single ubiquitin molecules can be conjugated to the target (monoubiquitination) or ubiquitin chains can be formed by linking individual ubiquitin molecules by seven internal lysines (K6, K11, K27, K29, K33, K48, K63) or amino-terminal methionine to form linear ubiquitin chains (polyubiquitination) [4]. Different ubiquitin chains are recognized by specific ubiquitin-binding domains [5]. This variety of ubiquitin modifications and ubiquitin-binding proteins is the foundation of selectivity of the ubiquitin system and allows the transmission of defined signals in a precise spatial and temporal manner. The various types of chains play a critical role in distinct cellular processes with K48, K63, and linear ubiquitin chains being the most extensively studied. K48-linked ubiquitin chains and mixed K11/K48-linked ubiquitin chains [6] generally induce degradation of the modified protein via the proteasome, a multisubunit complex that recruits and proteolyzes ubiquitin-modified substrates [5] (Fig. 1). This process is used during signaling events and in transcriptional regulation, but also plays a critical role in protein homeostasis [1]. K63-linked, linear chains or branched/mixed chains can form the scaffolding platform for further protein recruitment and signaling [7]. Cellular events mediated by ubiquitination are counteracted by ubiquitin hydrolases/deubiquitinating enzymes (DUBs), which can cleave specific ubiquitin linkages and/or remove ubiquitin modifications more generally [8] (Fig. 1). DUBs play a critical role in maintaining the ubiquitin system at an equilibrium, but also in restricting signaling to avoid pathway hyperactivation [9]. Given the importance of ubiquitination for a vast number of cellular processes and for overall organismal homeostasis, it is not surprising that this instrumental posttranslational modification has to be tightly controlled and regulated at many levels. In this review, we discuss the enzymes and targets in the ubiquitin system that regulate the fundamental processes of inflammation and cell death, as well as the pathophysiology resulting from their dysregulation. Finally, we highlight the role of targeted therapeutics in the inhibition of the proteasome in the inflammatory response, the interplay of immunomodulatory drugs (iMiDs) in the ubiquitin proteasome system (UPS), and the DUB-like enzyme PLpro whose activity plays a role in the pathogenesis of the topical SARS-CoV-2 pandemic.

### Ubiquitination in the regulation of inflammasomes and cell death

#### Ubiquitination in TNF-mediated signaling

**Fig. 2:**
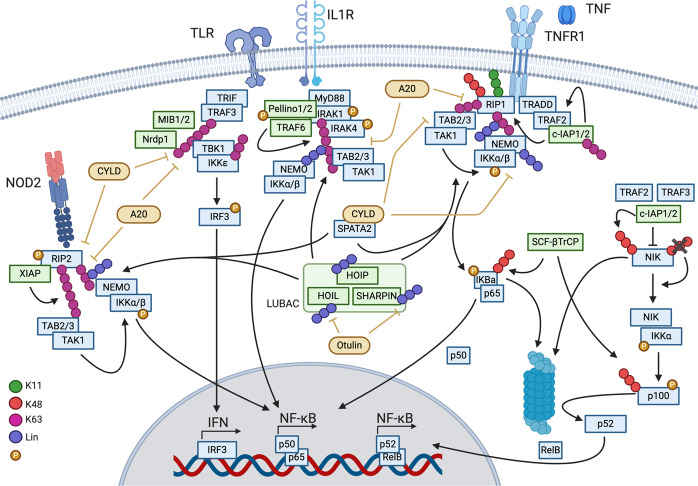
Ubiquitination in inflammatory signaling. Signaling mediated by TNFR1, Il-1R, TLR3/4, or NOD2 relies on complex ubiquitination involving multiple ubiquitin chains to activate inflammatory gene expression. Green indicates ubiquitin ligases and yellow deubiquitinase. Ubiquitin linkage types are indicated in the figure.

Ubiquitination plays a critical role during TNF (tumor necrosis factor)-mediated inflammatory signaling and cell death regulation. TNF is the most-studied member of the TNF family of inflammatory cytokines [10], and induces NF-κB (nuclear factor κ-light-chain-enhancer of activated B cells) and MAPK (mitogen-activated protein kinase) mediated gene expression, but also apoptotic and necroptotic cell death. Ubiquitination and deubiquitination of various components of the TNF-induced signaling complexes regulate the stability of these complexes and directly influence cellular fate. TNF binds to its cell surface receptor TNFR1 (TNF receptor 1) to trigger protein complex assembly, which is initially ubiquitin independent. TRADD (TNFRSF1A associated via death domain) and RIP1 (receptor interacting protein kinase 1; RIPK1) are recruited to the TNFR1-associated complex via homotypic death domain (DD) interactions [11, 12]. The N-terminal domain of TRADD allows the recruitment of TRAF2 (TNF receptor–associated factor 2) via its TRAF domain [13]. TRAF2 brings along c-IAP1/2 (cellular inhibitor of apoptosis) via the interaction of c-IAP BIR1 (baculovirus IAP repeat) domain and the c-IAP1/2 interacting motif of TRAF2 [14–17].

c-IAP1/2 are RING (really interesting new gene) domain-containing ubiquitin ligases that mediate K11-, K48-, and K63-linked ubiquitination of several components of the TNFR1 complex, including RIP1 and themselves [18–21] (Fig. 2). Ubiquitination of RIP1 on lysine 377 in human (376 in mice) was shown to be critical for NF-kB activation [22, 23] as well as embryonal development in mice [24–26]. Mutagenesis of K376 to arginine resulted in reduced NF-κB activation, reduced complex I formation, and increased cell death [24–26]. Besides the stabilization of RIP1 within the complex, c-IAP1 can promote RIP1 degradation by adding K48-linked ubiquitin chains resulting in reduced cell death potential by RIP1 [27]. Loss of either c-IAP1 or c-IAP2 does not result in developmental defects, but the combined loss is embryonic lethal and partially rescued by the loss of TNFR1 [28].

Another ubiquitin ligase, LUBAC (linear ubiquitin chain assembly complex) [29], is recruited to the complex by binding c-IAP1/2-generated K63-linked ubiquitin chains [30] (Fig. 2). LUBAC consists of SHARPIN (SHANK-associated RH domain interactor) [31–33], HOIP (HOIL-1-interacting protein) [29], and HOIL-1 (heme-oxidized iron-responsive element binding protein 2 ubiquitin ligase-1) [29], and it mediates the linear ubiquitination of proteins in signaling complexes. HOIP is the E3 responsible for the assembly of linear ubiquitin chains mediated by its RBR domain (RING in-between RING–RING) and the LDD (linear ubiquitin chain determining domain) regions [32, 34–36]. HOIP deficiency induces apoptosis resulting in embryonic lethality at E10.5, which can be rescued by the loss of TNFR1 to E17.5 [37]. HOIL-1 has an RBR domain as well, but its activity is not critical for the linear ubiquitination of LUBAC substrates [38]. A recent publication, however, showed that HOIL-1 E3 activity mediates monoubiquitination of LUBAC components thus forming a platform for the extension of autoinhibitory ubiquitin chains by HOIP [39]. Knockout of HOIL-1 in mice is embryonic lethal, but can be rescued by combined loss of caspase-8 and MLKL, demonstrating the clear interplay of cell death and ubiquitination [40]. The third component of LUBAC, SHARPIN, has no catalytic activity but it is critical for proper signaling and complex stability [33]. Mice with mutations in the SHARPIN gene (cpdm-mice) develop chronic proliferative dermatitis [41, 42], which can be prevented by the deletion of TNF, thus proving the critical role of functional LUBAC for TNF-mediated signaling [32]. Once LUBAC is recruited to the TNFR1 complex, it ubiquitinates several complex components including NEMO, RIP1, TRADD, and TNFR1 [32, 43, 44].

The key effectors of TNF-mediated prosurvival signaling are the kinase complexes IKK (IκB kinase) and TAK1/TAB2/3 (transforming growth factor β-activated kinase 1, TAK1-binding proteins 2/3), which are recruited in a ubiquitin dependent fashion. TAB2 and 3 bind to K63 ubiquitin chains via their carboxy-terminal zinc-finger (ZnF) domain and recruit TAK1 to the signaling complexes that activate NF-κB and MAPK pathways [45, 46]. The IKK complex consists of kinases IKKα/β and the adapter protein NEMO, which recruits the complex through binding of its LZ (leucine zipper) domain to K11 and K63 chains [23, 47] and via high affinity binding of its UBAN (ubiquitin binding in ABIN and NEMO proteins) domain to linear ubiquitin chains [48]. Linear ubiquitination further stabilizes signaling complexes and facilitates MAPK and NF-κB activation [44, 48]. The signaling platform created by K63-linked and linear ubiquitin chains brings kinase complexes into close proximity and enables the activation of IKKβ by TAK1 [49] leading to the phosphorylation of IκBα and NF-κB1. Phosphorylated IκBα undergoes degradative ubiquitination by SCFβ-TrCP (SKP1-cullin-F-box - Beta-transducin repeats-containing proteins), while NF-κB1 is partially degraded, also through the E3 activity of SCFβ-TrCP [50]. This results in free p65 and the NF-κB1 processing product p50, which then can translocate to the nucleus (reviewed by Zhang et al. [51]).

Besides playing a critical role during TNF-mediated signaling, cellular IAPs also regulate noncanonical NF-κB signaling. Together with TRAF2 and TRAF3, c-IAP1/2 form a complex that controls the stability of the kinase NIK (NF-κB–inducing kinase) [52, 53]. Without any stimulus, NIK is constantly ubiquitinated by the E3 activity of c-IAPs from this complex leading to its proteasomal degradation [54] (Fig. 2). Inflammatory pathway activation, such as stimulation of TRAF3-binding and some TRAF2-binding TNF family receptors (e.g., CD40, Fn14), leads to disruption of the TRAF2/TRAF3/c-IAP1/2 complex by inducing degradation and/or translocation of the complex components to insoluble cellular fraction [55–57]. This allows the accumulation of NIK leading to IKKα activation and phosphorylation of p100 [58]. p100 is then ubiquitinated and processed to p52 [59] resulting in noncanonical NF-κB signaling.

#### Deubiquitination in TNF signaling

The activity and stability of TNF-mediated prosurvival signaling can be restricted by ubiquitin hydrolases or deubiquitinases (DUBs). So far, three DUBs have been described with critical function within these signaling complexes: A20/TNFAIP3, CYLD (cylindromatosis), and Otulin (OTU deubiquitinase with linear linkage specificity) (Fig. 2). Their molecular details as well as phenotypes associated with altered protein function will be discussed below. Initially identified as a TNF/NF-κB target gene [60], A20 is recruited to the TNF receptor complex via ZnF motifs: ZnF4 binds K63-linked, while ZnF7 has a preference for linear ubiquitin chains [61–63]. A20 is also a ubiquitin-editing enzyme that can mediate ubiquitination (using its ZnF4) or deubiquitination (through its OTU domain) of signaling complex components (e.g., RIP1) [64]. Whole body deficiency of A20 results in a severe multiorgan inflammation, which is not surprising given its crucial role in many signaling processes [65]. In addition, extensive studies with conditional knockouts of A20 in mice further underline the importance of A20 during infections and in maintenance of homeostasis (summarized by Martens and van Loo [66]). Inactivation of A20 by mutagenesis of the DUB active site (C103A) or disruption of ZnF4 function (C609A, C612A) resulted in viable animals with no overt phenotype [67, 68]. In both cases, mutagenesis sensitized mice in inflammatory disease models, such as DSS-induced colitis [68], EAE (experimental autoimmune encephalomyelitis), or TNF-induced shock (SIRS) [67]. In contrast, mutagenesis of the ZnF7 domain (C764A, C767A) resulted in inflammatory arthritis [69, 70], and combined inactivation of ZnF4 and ZnF7 domains phenocopied A20 KO mice [69]. Polykratis et al. additionally showed that ZnF7 mutagenesis resulted in less linear chains in complex I [70] providing further evidence that A20 protects linear chains from degradation via its recruitment to modified RIP1 as described before by Draber et al. [43].

The other TNF signaling–associated DUB, CYLD, is a USP domain–containing DUB with specificity for K63-linked and linear ubiquitin chains [71, 72]. CYLD is recruited to the TNFR complex in a LUBAC-dependent fashion through interaction bridged by the adapter protein SPATA2 (Spermatogenesis Associated 2) [73–75]. CYLD can limit TNF-induced prosurvival signaling and promote cell death [76–78]. Accordingly, absence of SPATA2 provides protection against TNF-induced cell death [79]. Genetic disruption of CYLD function can lead to embryonic lethality, increased cancer formation or developmental defects, suggesting a complex role of CYLD in regulating multiple signaling pathways [80].

Otulin (FAM105B) is a DUB with high specificity for hydrolyzing linear ubiquitin chains [81]. Like CYLD, Otulin interacts with LUBAC [43, 82]. However, Otulin is not recruited to the TNFR1 complex; instead, it regulates TNF signaling indirectly by acting on LUBAC [43]. Indeed, studies with catalytic inactive Otulin (Otulin C129A) showed that Otulin plays a critical role in deubiquitinating LUBAC components thus regulating their stability [83]. Furthermore, Otulin inactivation reduces TNFR1 complex formation and increases cell death [83]. Loss of Otulin in the hematopoietic compartment resulted in a TNF-dependent inflammatory phenotype [84]. Otulin C129A/C129A embryos died at E10.5 with extensive cell death in placenta and yolk sac, which was rescued partially by inactivating TNF cell death effectors caspase-8 and RIPK3 [83]. These findings suggest that dysregulated linear ubiquitination triggers cell death and implicate LUBAC, Otulin, and associated proteins in the regulation of cell death activation and inhibition.

#### Ubiquitination during TNF-induced cell death

**Fig. 3:**
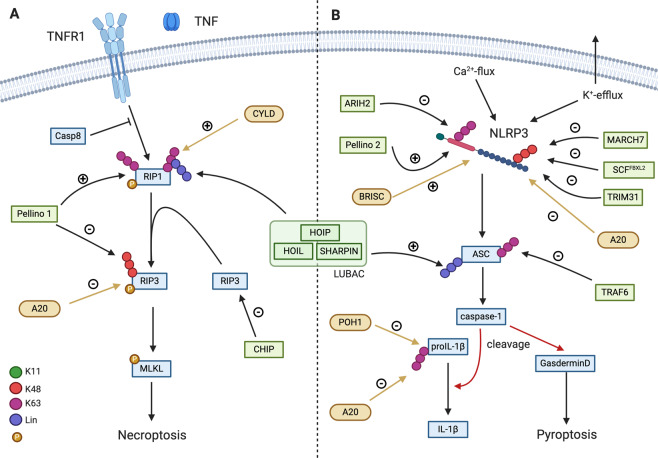
Ubiquitination in the regulation of cell death and inflammasome. Ubiquitination of the key components of TNF-stimulated cell death and NLRP3 inflammasome–mediated signaling regulate cell survival and inflammation. Green indicates ubiquitin ligases and yellow deubiquitinase. Ubiquitin linkage types are indicated in the figure.

Apart from mediating TNFR1-associated complex I assembly and MAPK and NF-κB activation, ubiquitination also plays a critical role in TNF-induced cell death complexes. These complexes include the cytosolic complex II and the necrosome, as well as the recently described bridging complex [85], which mediates the transition of RIP1 from complex I to the cytosol. This bridging complex consists of heavily ubiquitinated RIP1 and, based on caspase-8 activity, it can either induce RIP1 kinase–dependent apoptosis or necroptosis [85]. During necroptotic cell death, RIP1 undergoes ubiquitination with K63 and linear ubiquitin chains [86] (Fig. 3), with K115 as a prominent site of ubiquitination [87]. *Ripk1*^*K115R/K115R*^ mice did not show a developmental phenotype, but were more sensitive to TNF-induced systemic shock [26]. Although ubiquitin ligase(s) that modify RIP1 during necroptotic signaling are not well known, a study found that the E3 PELI1 (Pellino1) can promote RIP1 ubiquitination during necroptosis [88]. In addition to RIP1, PELI1 can also modulate RIP3 protein levels. Phosphorylation of RIP3 at T182 recruits PELI1 to promote K48-linked RIP3 ubiquitination on Lys363 leading to its proteasomal degradation thus restricting necroptosis [89]. Similarly, ubiquitin ligase CHIP (carboxyl terminus of Hsp70-interacting protein) can induce RIP3 lysosomal degradation and restrict necroptosis in a phosphorylation independent manner [89] (Fig. 3). Loss of RIP3 in vivo can partially rescue the phenotype observed in CHIP KO mice, suggesting that the absence of CHIP chaperone function can lead to cell death [90].

In addition to its role in proliferative signaling, A20 can directly influence necroptosis by deubiquitinating RIP3 at Lysine5, thus leading to reduced cell death [91]. Deubiquitination of RIP1 by CYLD in the necrosome was also suggested to facilitate necroptosis [92]. While it is clear that ubiquitination of complex II components take place during TNF-mediated cell death signaling, there is further research needed to fully understand its significance in regulating cell death and inflammatory responses.

#### E3 activity of IAPs in direct regulation of cell death signaling

IAPs have been studied initially due to their ability to inhibit caspases [93]. However, the inhibitory activity of IAPs can be counteracted by the mitochondrial protein SMAC [94, 95]. Once released from the mitochondria, SMAC binds BIR domains of IAPs thus blocking their inhibitory ability [93, 94, 96]. This finding has led to the development of a class of small molecules called IAP antagonists or SMAC mimetics [97]. Treatment with IAP antagonists causes a conformational change that opens the c-IAP structure and allows c-IAP RING domain dimerization and consequent activation of c-IAP1/2 E3 ligase activity leading to autoubiquitination and subsequent proteasomal degradation [98, 99]. The prompt activation of c-IAP E3 activity triggers initial RIP1 ubiquitination and NF-κB and MAPK activation [20]. However, proteasomal degradation of c-IAPs allows NIK stabilization and stimulation of the noncanonical NF-κB pathway [55, 100]. NF-κB and MAPK activation cause upregulation of TNF and trigger TNFR1 mediated signaling, which cannot promote RIP1 ubiquitination and prosurvival signaling in the absence of c-IAPs [18, 59, 100]. Instead, RIP1 will form the apoptotic complex with FADD/caspase-8 or complex with RIP3 to promote apoptotic or necroptotic cell death, respectively [20]. This is a great example of the link between exacerbated ubiquitination and cell death induction, and it represents a mechanistic platform for development of IAP antagonists for treatment of various human diseases: from cancer to HIV latency [96, 97, 101].

#### Pattern recognition receptor signaling and ubiquitination

The innate immune system must operate in an efficient and safe manner to maintain organismal homeostasis by recognizing invading pathogens and initiating the host defense mechanisms [102]. Important components of this defense mechanism are proteins that recognize microbe-associated molecular patterns (MAMPs), such as nucleotide-binding oligomerization domain (NOD)-like receptor (NLRs) family [103]. Among the NLR group, NOD1 and NOD2 are crucial for innate immune responses to some bacterial infections as they sense bacterial peptidoglycans and stimulate NF-κB and MAPK signaling resulting in the expression of proinflammatory cytokines and chemokines [103]. NOD2 is particularly relevant for human pathologies as mutations in the NOD2 locus are linked with inflammatory genetic diseases such as Crohn’s disease, early onset sarcoidosis, and Blau syndrome [104].

NOD2-mediated signaling relies on RIP2 (kinase receptor-interacting protein 2; RIPK2) [105], which recruits a number of signaling regulators to the NOD2-associated protein complex including several ubiquitin ligases and DUBs [106]. Several RIP2-modifying E3s have been reported including c-IAP1/2, XIAP (X-linked inhibitor of apoptosis), Pellino3, ITCH (Itchy E3 Ubiquitin Protein Ligase), TRAF6, and TRIM27 (Tripartite Motif Containing 27), but the critical E3 in NOD/RIP2 pathways is XIAP [106]. XIAP binds the kinase domain of RIP2 with its BIR2 domain to promote K63-linked ubiquitination of RIP2 [107] (Fig. 2). This enables LUBAC recruitment and linear ubiquitination of RIP2 resulting in the activation of NF-κB and MAPK to promote production of inflammatory cytokines and chemokines [108]. Interestingly, the kinase domain of RIP2 does not have enzymatic role in NOD2 signaling, only scaffolding, as it serves as a docking module that enables XIAP to bind and ubiquitinate RIP2 [109, 110]. Disruption of RIP2-XIAP binding blocks RIP2 ubiquitination and inhibits NOD2 signaling, demonstrating that XIAP-mediated RIP2 ubiquitination is critical in this inflammatory pathway [109]. Comparable to TNF signaling, the same trio of DUBs (A20, CYLD, and Otulin) removes K63-linked and linear ubiquitin chains from RIP2 thus restricting NOD2 proinflammatory signaling [108].

#### Inflammasomes

While some pattern recognition receptors (PRRs) recognize MAMPs, others are stimulated by damage-associated molecular patterns (DAMPs) leading to inflammasome activation and an inflammatory response (Fig. 3) [111]. Activated inflammasomes lead to catalytically active capase-1, which process the proinflammatory cytokines IL-1β and IL-18 [112, 113]. The most studied PRR is NLRP3 (nucleotide-binding and leucine-rich repeat-containing protein 3), which can be stimulated by a variety of DAMPs or MAMPs in a two-step process that involves priming (step 1) and subsequent activation (step 2) [114, 115]. Besides a cytokine response, inflammasome activation can lead to pyroptotic cell death. Pyroptosis is induced by caspase-1/11-mediated cleavage of GSDMD (gasdermin D), which separates the inhibitory and the lytic domains of GSDMD. The cell death inducing N-terminal domain forms membrane pores and allows spillage of cellular contents and ion flux [116, 117].

NLRP3 activity is regulated by various mechanisms. Its levels can be induced by the priming step [118] and ubiquitination can regulate its stability as well as its activity [119]. Several E3 ligases (TRIM31, SCFFBXL2, and MARCH7) can induce degradation of NLRP3 by K48-linked ubiquitination and subsequent proteasomal or autophagosomal degradation [120–122] (Fig. 3). The E3 ARIH2 (Ariadne homolog 2) inactivates NLRP3 by K63-linked ubiquitination without induction of degradation [123]. NLRP3 ubiquitination can also be stimulatory, as is the case with E3 Pellino2, which promotes K63-linked NLRP3 modification [124]. Ubiquitination of other inflammasome-associated proteins such as ASC (apoptosis-associated speck-like protein containing a CARD) can modulate their activity as well. TRAF6 directly or indirectly induces ASC degradation by K63-linked ubiquitin chains [125], while LUBAC can contribute to inflammasome activation by linear ubiquitination [126, 127]. In an analogous fashion to other signaling pathways, deubiquitination can also regulate inflammasome activity. The BRISC DUB complex components BRCC3 (BRCA1/BRCA2-containing complex subunit 3) and ABRO (Abraxas brother 1) are critical in mediating NLRP3 inflammasome activation by deubiquitination [128, 129]. In contrast, A20 activity dampens inflammasome activation although it likely does not act directly on NLRP3 but rather on multiple proteins whose activity and/or stability play important role in inflammasome activity (RIP1, RIP2, RIP3, IL1-β) [130, 131].

#### IL-1β and TLR signaling

IL-1β is one of the two major proinflammatory cytokines released during pyroptosis. It is activated by cleavage and released from the cell in a passive manner. Upon binding of IL-1β to IL-1 receptor, the adapter protein MyD88 (myeloid differentiation primary response 88) is recruited via TIR (Toll/interleukin-1 receptor) domain interaction [132, 133]. Through a series of homotypic DD–DD interactions, MyD88, IRAK1/2 (Interleukin-1 receptor–associated kinase), and subsequently IRAK4 are recruited to the complex. IRAK4 transactivates itself by autophosphorylation and also phosphorylates and activates IRAK1 [134]. E3s Pellino1/2 and TRAF6 can bind IRAK1, which activates Pellino1/2 by phosphorylation [135, 136] (Fig. 2). These ubiquitin ligases mediate K63-linked ubiquitination of the complex [137, 138], resulting in LUBAC recruitment as well as the activation of MAPK and NF-κB signaling via TAB2/3-TAK1 and the IKK complex (as described above). Signaling by LPS-binding receptor TLR4 (toll-like receptor 4) shares the same pathway components such as IL-1R signaling complex, and both TLR/IL-1R-mediated signaling events can be restricted by DUBs A20 and CYLD68, [139] (Fig. 2). In addition, IL-1β is also deubiquitinated by POH1 inhibiting its cleavage by caspase-1 [140] (Fig. 3). TLRs can form a different kind of intercellular complex. The TIR domain of TLR4 can recruit RHIM domain–containing adapter TRIF, which allows the recruitment of a different subsets of signaling mediators leading to MyD88-independent NF-κB signaling via RIP1 and TRAF6 [141, 142]. During TLR4-mediated necroptotic cell death, TRIF can recruit RIP3, and with it MLKL, therefore enabling RIP3 and MLKL ubiquitination [143]. The pathway is further activated by E3 ligases such as TRIM56, TRIM62, and Pellino, or it can be restricted by TRIM38 (summarized by Ullah et al. [144]). TRIF may also activate the TBK1 (tank-binding kinase 1) and IKKε to activate the transcription factor IRF3, resulting in the induction of Type 1 interferon response [145, 146]. K63-linked polyubiquitination of TBK1 is required for the induction of Type 1 interferons that is mediated by the E3s MIB1 (Mind Bomb 1), MIB2, and Nrdp1, whereas the DUBs CYLD and A20 reverse TBK1 ubiquitination and dampen Type 1 interferon signaling [147] (Fig. 2).

#### Pathologies associated with inflammatory pathway components

Mutations in E3s and DUBs participating in inflammatory pathways have been associated with various disease. Besides causing autoimmune and autoinflammatory diseases, mutations in these E3s/DUBs can be found in various types of cancers due to the prosurvival signaling function of the pathways. CYLD was first identified as a gene mutated in cylindromatosis, a hereditary form of skin cancer [148], due to dysregulation of NF-κB signaling [76]. A20 seems to have a dual role in cancer. In some cancers, A20 loss of function mutations were identified, suggesting a tumor suppressive role, while high expression of A20 was associated with poor survival in other types (summarized by Martens and van Loo [66]). Copy number gains of c-IAP1/2 have been described in diffuse large B cell lymphoma [149] and carcinomas [150], indicating a role as mediators of TNF-mediated NF-κB signaling. Moreover, deletions of c-IAP1/2 or their adapters TRAF2/3 were described in patients with multiple myeloma that lead to increased noncanonical NF-κB signaling [151, 152]. The IAP family member XIAP, and inherited loss of XIAP, has been implication in X-linked lymphoproliferative type 2 disorder (XLP2), which manifests with lymphohystiocytosis, hypogammaglobulinemia, and lymphomas [153].

Besides their roles in oncogenesis, several TNF pathway components have been associated with autoinflammatory diseases in patients. Haploinsufficiency of A20 was identified in multiple studies resulting in inflammatory diseases marked by hyperactivated NF-κB signaling [154] (for detailed summary [66]). Mutations in Otulin were identified in three patients suffering from autoinflammatory disease (named ORAS or OTULIN-related autoinflammatory syndrome) manifested by panniculitis and recurrent fever [84, 155]. Analysis of one mutation showed reduced protein stability leading to dysregulation of TNF signaling and sensitization to TNF-induced cell death [155]. Treatment of patients with ORAS with anti-TNF antibodies resulted in a decrease of measured inflammatory parameters such as CRP or neutrophil counts [84]. Mutations in the LUBAC components HOIP and HOIL-1 have also been associated with inflammatory phenotypes in human patients [156–158]. Fatal HOIL-1 mutations were identified in three patients of two families, resulting in systemic autoinflammation and susceptibility to infections [157]. Mutations in HOIP leading to loss of expression were associated with multiorgan autoinflammation and immunodeficiency [156, 158]. All described HOIL-1 or HOIP mutations lower the expression of all LUBAC components, which speaks to the critical role of all components for the complex stability. Furthermore, it is increasingly apparent that dysregulation of linear ubiquitination affects many critical signaling events and leads to inflammatory diseases.

### Therapeutic modulation of the ubiquitin system

In addition to the IAP antagonists described above, we highlight proteasome inhibitors, immunomodulatory compounds, and SARS-CoV2 protease inhibitors as emerging strategies to target the ubiquitin/proteasome enzymes and pathways featured in this review.

### Proteasome inhibition and inflammation response

The proteasome is an important mediator of proliferative response in tumor and immune cells by regulating NF-κB signaling. More specifically, the proteasome participates in the activation of the canonical NF-κB pathway by degrading the negative regulator IκBα to release the transcriptionally active p50 and p65 heterodimer, and in the non-canonical NF-κB pathway, by proteolytically processing the active p52 NF-κB subunit from the longer precursor p100 (Fig. 2) [51]. The clinically approved inhibitors of the b5 proteasomal protease are bortezomib, carfilzomib, and ixazomib, which have multifaceted antiproliferative effects, including inhibition of NF-κB signaling, and have shown the most clinical benefit in multiple myeloma [159].

The clinical benefit of proteasome inhibitors in multiple myeloma was originally proposed to be a consequence of the genetic aberrations that activate NF-κB signaling in multiple myeloma cells, for example as mentioned above [151, 152], that impose NF-κB-dependence [160]. That said, bortezomib was also shown to paradoxically activate NF-κB signaling in multiple myeloma cell lines, primary multiple myeloma cells, and in xenografts, indicating that bortezomib-induced cytotoxicity in multiple myeloma cells is not fully attributable to inhibition of NF-κB signaling [161, 162]. However, the more selective proteasome inhibitors carfilzomib and ixazomib have demonstrated NF-κB repression in multiple myeloma cells [163, 164]. This inhibitory activity reduces the expression of NF-κB–induced cytokines and growth factors that regulate multiple myeloma cells and also pre-osteoclasts, resulting in less bone destruction [165–167].

Proteasome inhibition also exhibits multiple effects on the host immune cells. At clinical doses (≤20 nM), bortezomib treatment increases the levels of immunostimulatory cytokines (IL-2, IL-12, IL-15) in lymphocytes, stabilizes their receptors, and increases their effector function against tumor cells [168]. Bortezomib was also shown to decrease expression of HLA class 1 molecules in multiple myeloma cells and sensitize them to NK cell–mediated lysis [169]. In addition, bortezomib treatment upregulates immunostimulatory tumor antigens, priming the cancer cells for NK cell toxicity. [170]. These studies have shown that bortezomib treatment mediates a dual antitumor effect, by inhibiting tumor cell proliferation and by increasing sensitivity to cytotoxic immune cells. Therefore, immunotherapeutic strategies with antitumor T cell and/or NK cell could provide a combinatorial benefit with bortezomib treatment. In agreement, bortezomib is reported to synergize with the apoptotic potential of cytokines in tumors [171].

### Immunomodulatory drugs and the ubiquitin proteasome system

The iMiDs, such as thalidomide, are a class of compounds that display potent anti-inflammatory, antiangiogenic, antiproliferative, and immunomodulatory effects. One of the defining characteristics of the iMiDs is their ability to inhibit the LPS-induced production of the proinflammatory cytokine TNF-α [172], and iMiDs are capable of rescuing mice from endotoxic shock [173]. Similarly, iMiDs reduce the production of IL12 [174], but IL6 is unaffected [172]. In the production of cytokines induced by the MYD88-independent TLR4 pathway, translocation of the transcription factor IRF3 (interferon regulatory factor 3) to the nucleus following its phosphorylation is critical in the regulation of the IFN-1 response [145]. It has been shown that iMiDs prevent this translocation, leading to a reduction in the IFN-1 response [175]. While it is known that IRF3 is tightly regulated by polyubiquitination and subsequent proteasomal degradation [145], how this process is directly or indirectly affected by iMiD treatment is unknown.

Thalidomide, and the structurally related iMiD analogs lenalidomide and pomalidomide, have been shown to harness ubiquitin machinery through recruitment of neo-substrates to the ubiquitin ligase complex CRL4^CRBN^ (CUL4-RBX1-DDB1-CRBN) for ubiquitination and subsequent proteasomal degradation. The clinical efficacy of lenalidomide and pomalidomide in treating multiple myeloma is linked to the degradation of the transcription factors IKZF1/3 (Ikaros zinc finger proteins 1 and 3), as well as the accompanied IL2 upregulation [176–179]. In addition, lenalidomide uniquely induces the degradation of CK1α (casein kinsase 1α) and leads to synthetic lethality in del(5q) myelodysplastic syndrome [180]. A more recent study revealed that the “molecular glue” feature of iMiDs induces ternary complex formation between cereblon (CRBN) and a number of Cys2-His2 (C2H2) ZnF proteins, promoting their subsequent ubiquitination and degradation [181]. This structural plasticity of CRBN has inspired the development of next-generation iMiDs (known as CRBN modulators) and heterobifunctional protein degraders [182, 183]. It remains to be explored whether the degradation of non-IKZF1/3 targets may contribute to the therapeutic activities of established and novel iMiDs.

While the antitumor effects of iMiDs are dependent on CRBN [184], CRBN-independent activity accounts for 30–40% TNF-α inhibition following TLR4 activation [172, 175]. IRF3 translocation and resultant IFN-1 signaling has been shown to proceed in the absence of CRBN in murine studies, which at least partly accounts for the described CRBN-independent activity [175]. The remainder of the anti-inflammatory activity displayed by iMiDs may therefore be CRBN-dependent. It is possible that iMiDs may similarly recruit key proteins that regulate inflammation to the CRL4^CRBN^ complex to promote their proteasomal degradation.

### Modulation of the immune response by the SARS-CoV-2 protease PLpro

SARS-CoV-2 is the human coronavirus responsible for the COVID-19 global pandemic, and a defining factor of SARS-CoV-2 pathogenesis is the inhibition of innate immune sensing and reduced interferon response [185, 186]. CoV-2 PLpro, the papain-like protease of SARS-CoV-2, is required for viral polyprotein cleavage and is thus essential for viral replication [187]. In addition, CoV-2 PLpro cleaves both ubiquitin and the ubiquitin-like protein ISG-15 from host proteins, thereby displaying DUB and de-ISGylase activity [188]. One notable target is IRF3, which is a critical component in the type I interferon pathway activated by TLR4 as previously discussed in the context of iMiDs [146]. ISGylation of IRF3 typically provides an activating role by shielding IRF3 from ubiquitylation and subsequent degradation, allowing its phosphorylation and nuclear translocation [145]. Heterologous expression of PLpro within mammalian cells, or by infection with SARS-CoV-2, is reported to decrease phosphorylation of both IRF3 and its activator; TBK1. TBK1 phosphorylation is also an activator of the NF-κB pathway, causing upregulation of inflammatory signaling [189]. Concordantly, INF-β and NF-κB expression levels following poly(I:C) and TNF-α treatments, respectively, were also significantly decreased by infection or PLpro expression [190]. This shows a clear role for the DUB/deISGylation activity of PLpro in the suppression of both IFN and NF-κB signaling pathways, and may contribute to the pathogenesis of the virus.

The effects of CoV-2 PLpro on the IFN and NF-κB signaling pathways, which facilitate establishment of viral infection, as well as being essential for viral replication, make it a promising target for therapeutics. A number of so-called naphthalene-based inhibitors, originally developed against PLpro enzyme of SARS-CoV responsible for the 2003 SARS outbreak [190–193], have shown biochemical and cellular activity against CoV-2 PLPro, owing to the two proteins’ high homology (82% sequence identity). Two related scaffolds in this class are exemplified by GRL-0617 [190] and 3k [194], which both inhibit purified CoV-2 PLpro and prevent viral replication in cells. Interestingly, inhibitors in this series occupy the S4/S3 binding pockets, which are located away from the catalytic triad but block the entry of the ISG-15 C-terminus to cause inhibition. Two newly developed peptide-based inhibitors of CoV-2 PLpro, VIR250 and VIR251, mimic the sequence of PLPro cleavage sites and irreversibly bind to the catalytic Cys111 [195]. While these compounds have not been investigated for antiviral activity in cells, VIR251 showed a clear inhibition of the DUB/deISGylation activity of CoV-2 PLpro.

## Conclusions

The ubiquitin system is complex, multifaceted, and is crucial for the modulation of a vast number of cellular processes. Ubiquitination is tightly regulated by a number of enzymes including E1s, E2s, E3s, and DUBs, and dynamically regulates inflammation and a number of programmed cell death mechanisms. Notably, the TNF signaling pathway is controlled by competing ubiquitin conjugation and deubiquitination that governs both proteasomal degradation and signaling complex formation. Aberrant ubiquitin regulation of inflammatory pathways is reported in a number of pathologies including cancers and autoinflammatory diseases. Ubiquitination is capable of both activating and dampening inflammasome activation through the control of either protein stability, complex formation, and, in some cases, directly affecting receptor activity.

Targeting dysfunctional aspects of the ubiquitin system is a promising approach in the treatment of inflammatory disease, cancers, and infectious disease. Proteasome inhibitors decrease tumor cell proliferation, reduce osteoblast activity, and promote sensitivity to immune cells. Although the antiproliferative effects of proteasome inhibitors are complex and not fully understood, inhibition of the NF-κB signaling pathway appears to play a key role. iMiDs co-opt the UPS by recruiting neosubstrates to the ubiquitin ligase complex CRL4^CRBN^, thus directing their degradation, and have been designed to show promising specificity. The mechanisms underlying the therapeutic benefits of IMiDs are also complex, as their anti-inflammatory activity is partially CRBN-independent, and their regulation of IRF3 signaling remains to be fully elucidated. Finally, the novel coronavirus SARS-CoV-2 modifies the TLR4-induced production of host cytokines through the DUB/deISGylating activity of its papain-like protease PLPro. Pre-clinical inhibitors of PLpro reverse this effect and attenuate viral replication, thus PLPro may be a therapeutic target to combat SARS-CoV-2 morbidity and mortality, and potentially future coronaviruses that utilize similar mechanisms. Looking forward, future work that deepens our understanding of the complex cellular role of the ubiquitin system, including its role in disease processes, is vital. Such work could lead to the identification of novel targets and efficacious therapeutics to treat the wide array of diseases in which the ubiquitin system is implicated.
